# AllelicImbalance: an R/ bioconductor package for detecting, managing, and visualizing allele expression imbalance data from RNA sequencing

**DOI:** 10.1186/s12859-015-0620-2

**Published:** 2015-06-12

**Authors:** Jesper R. Gådin, Ferdinand M. van’t Hooft, Per Eriksson, Lasse Folkersen

**Affiliations:** Atherosclerosis Research Unit, Karolinska University Hospital Solna, Center for Molecular Medicine, Bldg L8:03, S-171 76 Stockholm, Sweden; Center for Biological Sequence Analysis, Department of Systems Biology, Technical University of Denmark, 2800 Lyngby, Denmark

**Keywords:** Allelic imbalance, Allele-specific expression, RNA sequencing, Gene expression, SNP

## Abstract

**Background:**

One aspect in which RNA sequencing is more valuable than microarray-based methods is the ability to examine the allelic imbalance of the expression of a gene. This process is often a complex task that entails quality control, alignment, and the counting of reads over heterozygous single-nucleotide polymorphisms. Allelic imbalance analysis is subject to technical biases, due to differences in the sequences of the measured alleles. Flexible bioinformatics tools are needed to ease the workflow while retaining as much RNA sequencing information as possible throughout the analysis to detect and address the possible biases.

**Results:**

We present AllelicImblance, a software program that is designed to detect, manage, and visualize allelic imbalances comprehensively. The purpose of this software is to allow users to pose genetic questions in any RNA sequencing experiment quickly, enhancing the general utility of RNA sequencing. The visualization features can reveal notable, non-trivial allelic imbalance behavior over specific regions, such as exons.

**Conclusions:**

The software provides a complete framework to perform allelic imbalance analyses of aligned RNA sequencing data, from detection to visualization, within the robust and versatile management class, ASEset.

**Electronic supplementary material:**

The online version of this article (doi:10.1186/s12859-015-0620-2) contains supplementary material, which is available to authorized users.

## Background

Regulatory variants that alter gene expression can be examined, based on allelic imbalance (AI), i.e., alleles can be differently expressed in an individual if the regulatory region around a gene differs. In RNA sequencing data, it is possible to determine the allele from which a specific read originates when there is at least one heterozygous SNP in the sequence read [1]. An AI event indicates that there is a variant that changes gene expression within or near that gene. It only takes one individual, assuming that there is a heterozygous site in the gene of interest.

The detection of an AI event is not trivial, comprising several steps, including library preparation [2], sequencing [3], mapping [4], and analysis of somatic mutations and RNA-editing [5], which can bias the allele count. To counter such biases when determining the true AI for an exon or gene, a smaller region must be visualized to discover inconsistent patterns.

**Fig. 1:**
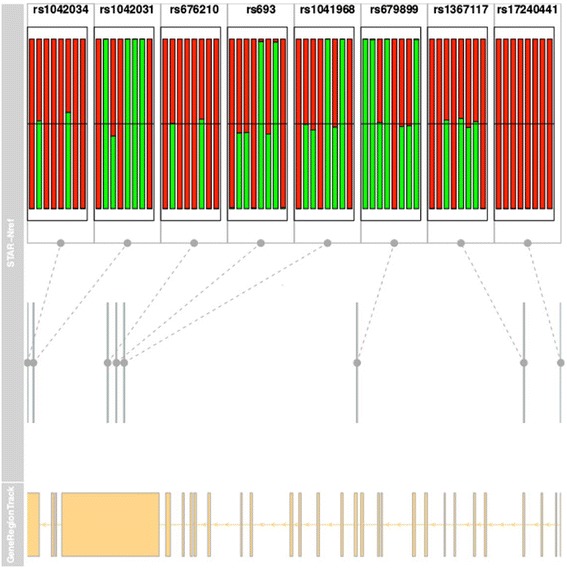
Short Title: AI consistency using glocationplot. Detailed Legend: On top are the fractions of alleles over *APOB* for SNPs with a MAF > 0.1. Each bar represents one of eight samples, and the grey lines in the middle show the SNP locations in *APOB* beneath in yellow. All SNPs shown are close around the black line, denoting 1:1 expression of the alleles. See Additional file 3 for the total allele count for each SNP

The AllelicImbalance package was developed to address these issues, allowing the user to test AI at a single gene or SNP quickly. Nevertheless, the package is suitable for performing any custom global AI analysis, because there is always a counting step and the need to store counts in a smart container, which facilitates access to custom requests from the user. For genes that have more than one heterozygous SNP and at least one sample, there is a function to visualize AI consistency easily over the gene as an internal validation to select SNPs that are suitable for further AI QTL study (Fig. 1). The package is easy to use, comprising an infrastructure that is linked to the Bioconductor environment, and allows the user to pose genetic questions quickly.

AllelicImbalance was developed to provide usability for inexperienced as experienced R-users. For inexperienced users, there is a standard protocol to create an ASEset from bam files, and functions, such as *barplot*, can be used directly on that ASEset class object; experienced users can customize nearly any part of the workflow. The design is based on RNA-sequencing, but AllelicImbalance can be used with any allele that is focused on a count-based technique, such as digital qPCR [6].

## Implementation

### Management

**Fig. 2:**
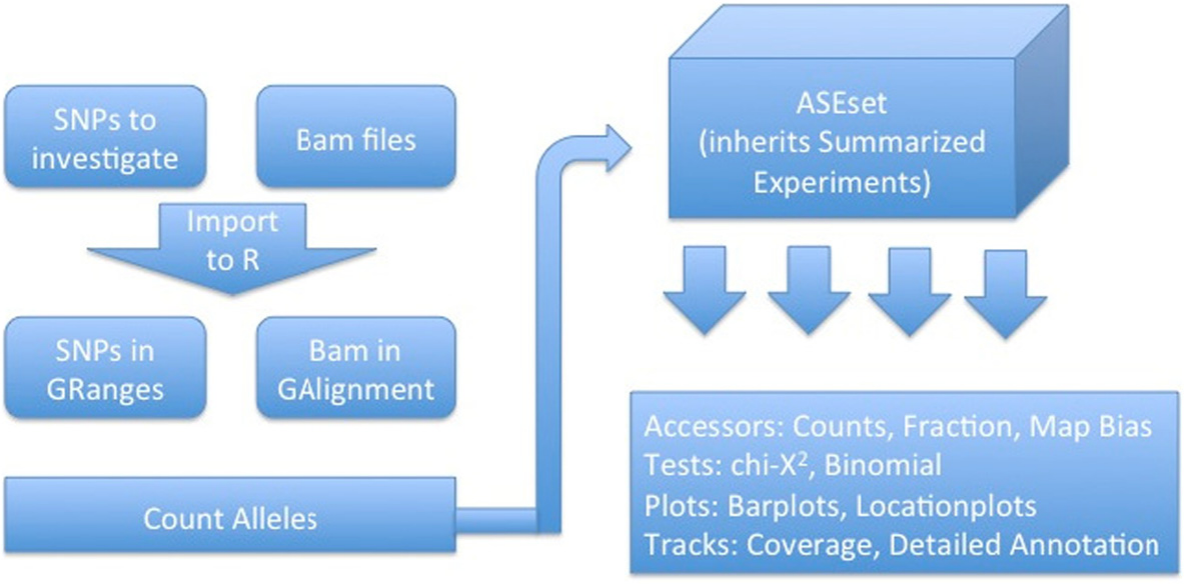
Short Title: Flowchart of a typical workflow in the AllelicImbalance package

**Fig. 3:**
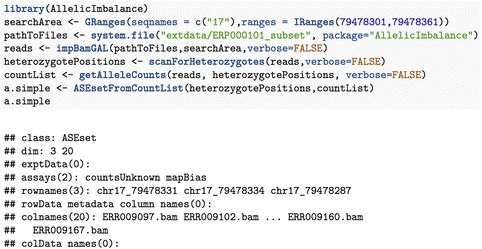
Short Title: A few simple commands are needed to construct an ASEset-class object. Detailed Legend: If the bam files are unprocessed before being imported into R, we recommend elaborating the filtering on the mapping with regard to quality and perfect mate-pairs before counting the alleles

ASEset is a new object class that summarizes sequencing data (see Fig. 2 on how to create one). It contains allele counts, phenotypes, and SNP positions and inherits the SummarizedExperiments class and all functions that can be applied to that class, such as subset and range operation [7]. The class has support for strand- and nonstrand-specific data. The first step in AI analysis is to create an ASEset from mapped data in bam file format and a set of SNPs of interest (see Fig. 3). The support functions will summarize the allele counts for each SNP rapidly and save them in an ASEset object.

### Detection

Equal amounts of reads are expected from two alleles, but one allele might be read more than the other by chance. A greater number of reads improves the estimate of the total distribution. Statistical tests, such as the chi-square and binomial tests, generate the probability that an observed difference is due to this sampling bias. These relatively simple and general tests can be applied directly to ASEset objects and return a matrix with p-values for each SNP and sample. The user can easily apply other custom tests by taking advantage of the ASEset accessor methods to retrieve allele fractions or counts, for example.

### Visualization and annotation

**Fig. 4:**
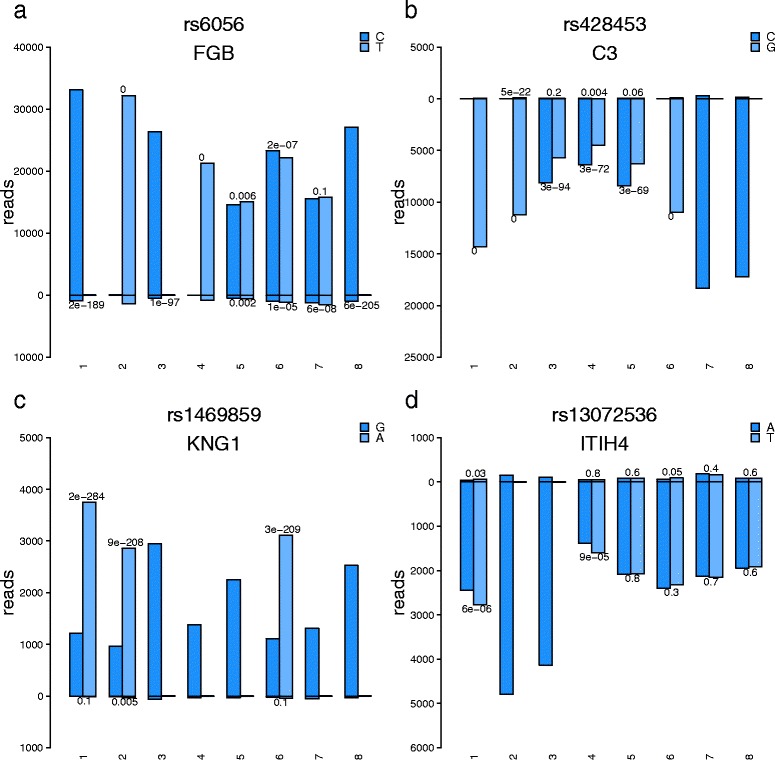
Short Title: The dual-strand barplot. Detailed Legend: The barplot from the AllelicImbalance package shows the number of reads aligning to each allele and strand for one SNP. Upward bars indicate the (+) strand, and downward bars indicate the (−) strand. The numbers under the bars are the p-values from testing whether a difference in allele expression is due to chance. Because the data are strand-specific, nearly all reads over this SNP are mapped to one strand, consistent with the location of the investigated genes. All samples in this figure are from liver. **a** Of the heterozygote individuals 5–7, 5 and 7 show no AI, whereas individual 6 shows significant AI. But, the plot also shows expression off of the opposite strand, which might comprise antisense transcripts. **b** Individuals 3, 4, and 5 show AI. **c** Individuals 1, 2, and 6 show strong AI. **d** Individuals 1 and 4 show AI, whereas subjects 5, 6, 7, and 8 show no AI. See Additional file 1 and 2 for barplots using different aligners

AllelicImbalance has good visualization capabilities and provides a rich description of allele-specific expression in a region. The *barplot* function (Fig. 4) has options to display the data as a fraction or count plot and can be used with the Bioconductor AnnotationDbi and GenomicFeatures packages to show the annotation of a gene, an exon, and transcript information [7].

**Fig. 5:**
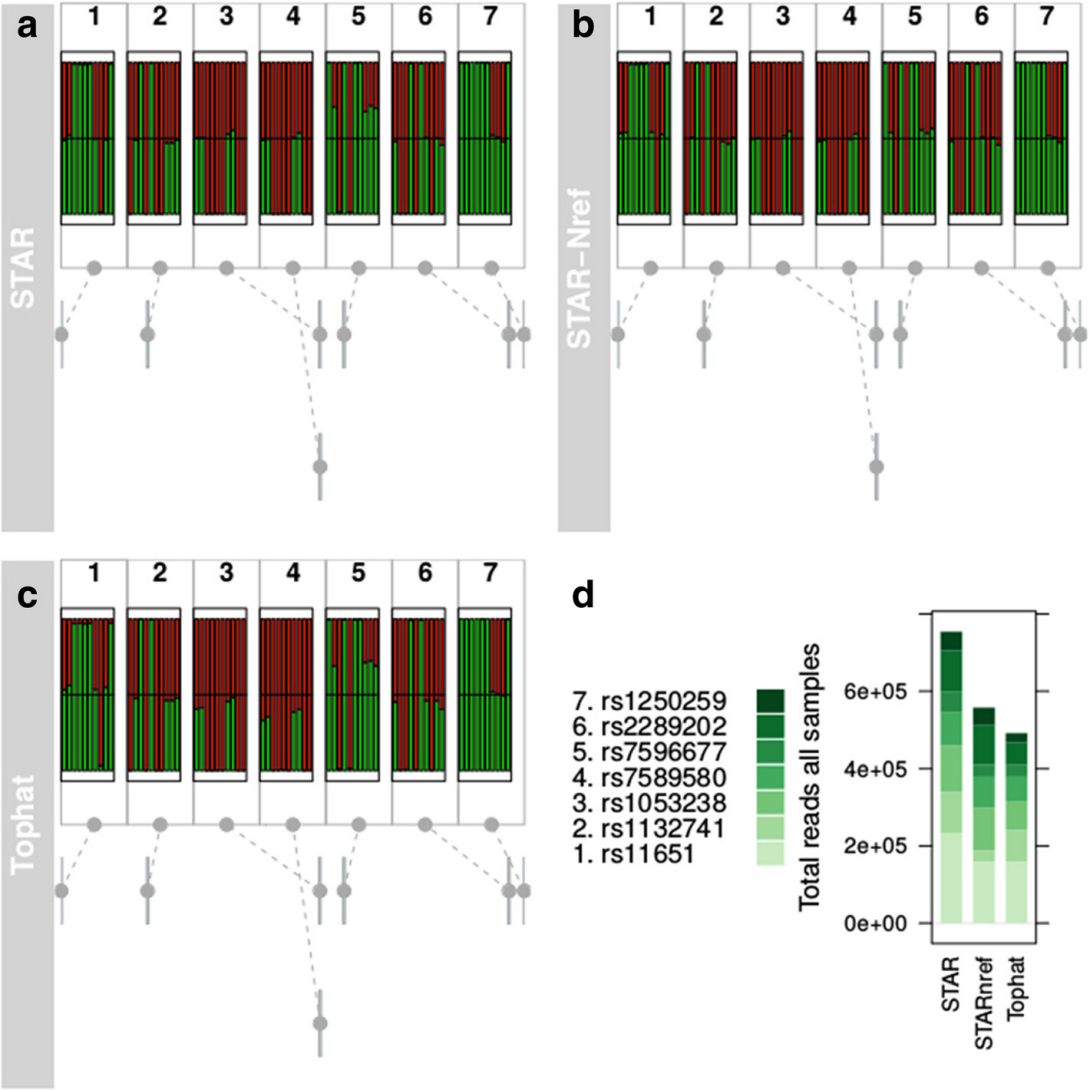
Short Title: AI consistency for different alignment methods for *FN1*. Detailed Legend: A comparison of fractions over SNPs between **a** STAR, **b** STAR with N-replaced SNP reference genome, and **c** TopHat2. In the normal STAR and TopHat2 run, the fraction lies around 1:1 for most SNPs, except SNP 5 (*rs7596677*), which shows strong AI. In **c**, however, the fractions are approximately 1:1 for all SNPs. **d** This graph summarizes the total counts for each SNP over all samples for the alignment methods. See Additional file 4 for the total allele count for each SNP

The bioconductor package Gviz [8] uses tracks and trellis graphics to imitate genome browsers’ visualization of a genomic region [9]. To integrate AllelicImbalance data as a track, it takes merely a function call over an ASEset object to create an object that is directly applicable for use with Gviz. The most common applications of these tracks have been wrapped in a function, called *glocationplot*. The g*locationplot* function displays several barplots in the same graph and marks their location in a region (Figs. 1 and 5).

### Mapping bias

An RNA sequencing read that contains SNPs can lead to a mapping bias—eg, reads that are more similar to the reference will map more often. This bias must be measured in the alignment step, such as through the generation of artificial reads that are equally distributed for both alleles over each SNP of interest [4]. In the alignment of reads, it is also possible to allow for more mismatches to decrease the bias toward the reference allele, but this step could affect the accuracy of the mapping [10].

Alignment to personal phased genomes is another method to handle mapping bias, requiring DNA sequencing of the same individuals [11] or ultimately personal transcriptomes, necessitating longer RNA sequencing reads [12]. To this end, AllelicImbalance has a function that defines the expected allele ratios other than 1:1 to adjust for this mapping bias when searching for AI. The package also has a function that creates a reference genome in which known SNPs are masked by the generic nucleotide N [13], which can then be used in a realignment. In this article, we reduced the mapping bias effect using this method and masked all known variants in dbSNP build 138 [14] prior to alignment.

## Results and discussion

AllelicImbalance can detect AI from RNA sequencing data that originate from transcriptional material. With sufficient read depth over a gene, it is even possible to detect and quantify the alleles in introns of the precursor mRNA. For example, we analyzed unpublished, strand-specific RNA sequencing data from the livers of 8 individuals and the aortas of 10 subjects (~90 million read-pairs each). To exemplify how AI can be used in a simple QTL analysis, four genes with high coverage—*FGB*, *C3*, *KNG*, and *ITIH4*—were plotted as dual barplots (Fig. 4). The plots show the binomial test p-values and provide visual confirmation of the presence of AI events. In this example, AllelicImbalance demonstrates that there are cis effects for several individuals in all of these genes.

Using established methods, such as eQTL, it would not have been possible to detect this effect in a limited sample size. In all of the exemplified genes, most reads came from one strand, suggesting that the interference of lncRNAs, for example, is low. But, at least 40 % of human genes are transcribed in both directions [15], potentially affecting the measurements of AI for a gene if there is AI on its antisense transcript.

To compare loci or individuals in which the read depth differs, it can be convenient to plot alleles as a fraction and inspect a wider region of all heterozygous SNPs, for example, of the same gene. Without interference from allele-specific splicing, we expect all SNPs over a gene to show the same pattern of fractions. Figure 1 shows an example for which there is consistency between heterozygous SNPs in a gene; there is no AI, but the overall 1:1 expression supports that the AI measurements are consistent in the RNA-seq data.

To illustrate the reduction in mapping bias, we replaced the SNPs in the reference genome with the generic nucleotide indicator N. All SNPs in dbSNP build 138 were masked in this manner, and we then reperformed the alignment with STAR. Figure 5 shows an example of how such steps can improve the detection of true AI compared with a default run using STAR (version 2.3.0) [16] or TopHat2 (version 2.0.4) [17].

## Conclusions

The AllelicImbalance package will be valuable in examining the genetics of RNA sequencing experiments. This software is a novel tool in the Bioconductor environment, in which no infrastructure that can perform AI analyses exists. The import functions are essential when retrieving allele counts for specific nucleotide positions from all RNA-seq reads. Similarly, the statistical analysis and plotting functions are necessary to identify any allele-specific expression patterns in one’s data. With merely a limited amount of samples, strong genetic effects on gene expression can be discovered.

## Availability and requirements

GPL3-licensed and available in the Bioconductor framework.

- Project name: AllelicImbalance software
- Project home page: http://www.bioconductor.org/packages/release/bioc/html/AllelicImbalance.html
- Operating system(s): Linux, Mac OSX, Windows
- Programming language: R
- Other requirements: None
- License: GPL3
- Any restrictions to use by nonacademics: GPL3

## Additional files

Additional file 1: Figure A1-A4. The corresponding barplots to figure 2 for a STAR alignment. Barplots for a TopHat2 alignment. Comparison between STAR, STAR dbSNP-masked reference and TopHat2 for AI fraction consistency in the APOB gene. A glocationplot for the FN1 gene with transcript annotation.

Additional file 2: **Includes counts, fractions and binomial test p-values for all individuals, rsids and alignment methods.**

Additional file 3: **Includes total counts over all samples for each SNP for the alignment methods for APOB.**

Additional file 4: **Includes total counts over all samples for each SNP for the alignment methods for FN1.**
