# Supplementary material for: AllelicImbalance: an R/ bioconductor package for detecting, managing, and visualizing allele expression imbalance data from RNA sequencing
Source: BMC Bioinformatics. 2015 Jun 12;16(1):194. doi: 10.1186/s12859-015-0620-2 (PMC4465016; doi:10.1186/s12859-015-0620-2)
Supplement: Supplementary file 1 — The corresponding barplots to figure 2 for a STAR alignment. Barplots for a TopHat2 alignment. Comparison between STAR, STAR dbSNP-masked reference and TopHat2 for AI fraction consistency in the APOB gene. A glocationplot for the FN1 gene with transcript annotation. [file 12859_2015_620_MOESM1_ESM.pdf]

Additional Figures

Figure A1

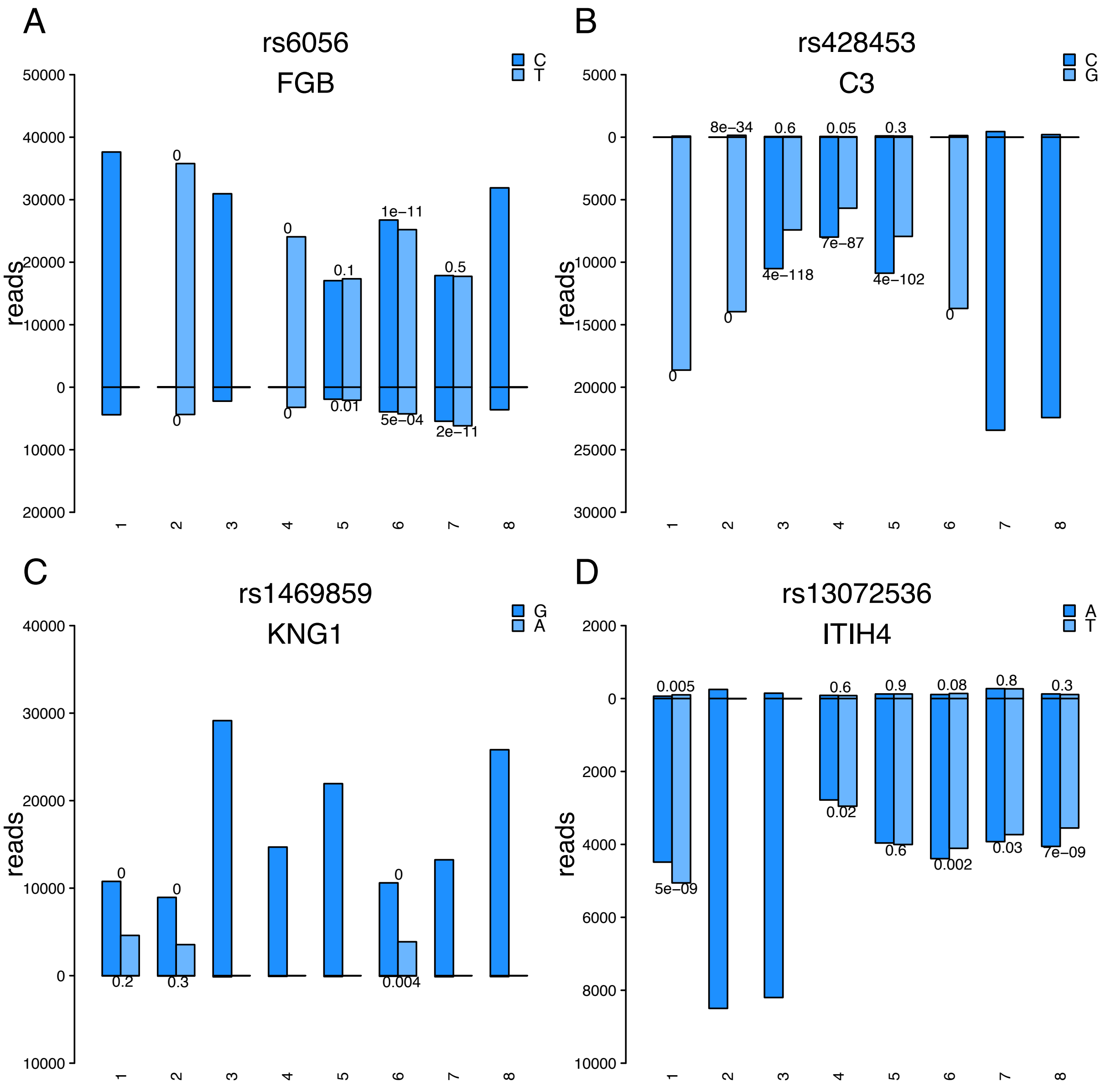

Figure A1. The corresponding barplots to Figure 2 for the alignment with default STAR.

Figure A2

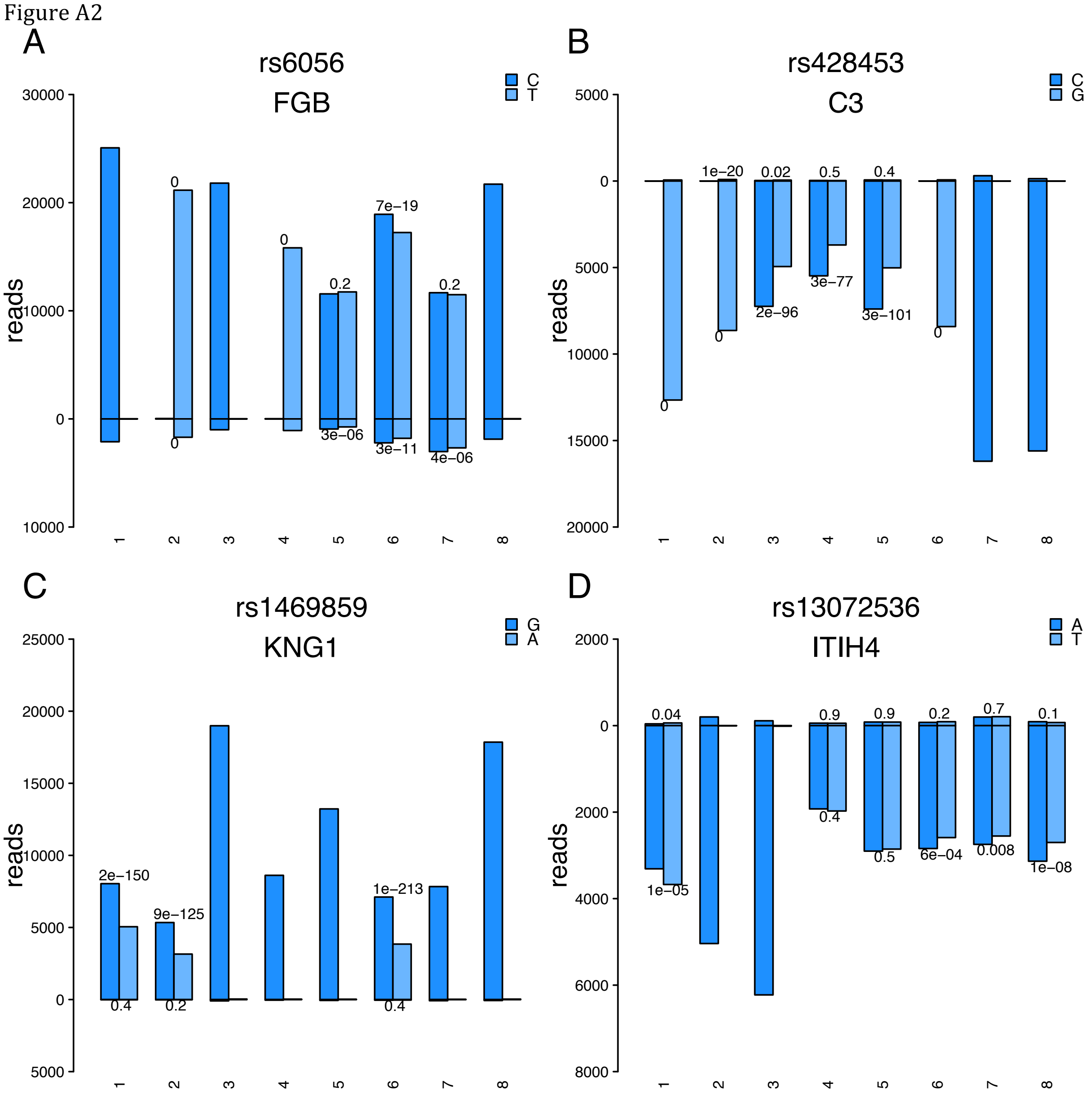

Figure A2. The corresponding barplots to Figure 2 for the alignment with default TopHat2 .

Figure A3

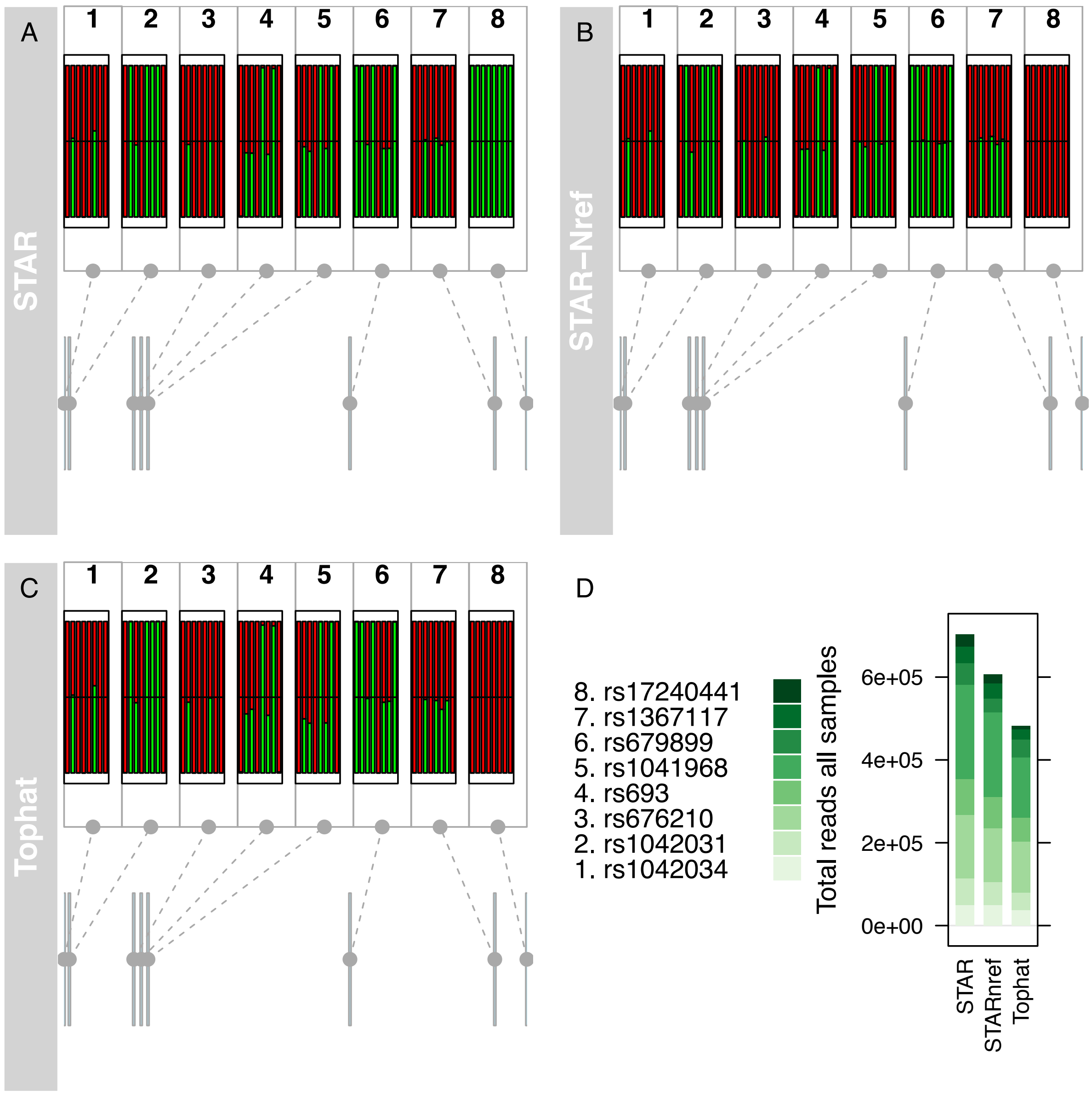

Figure A3. For the *APOB* gene a comparison of allele fractions for several alignment methods: A) STAR, B) STAR with an dbSNP-masked reference genome, and C) tophat. D) Summary of total count of reads for all eight samples that overlap with the SNPs.

Figure A4

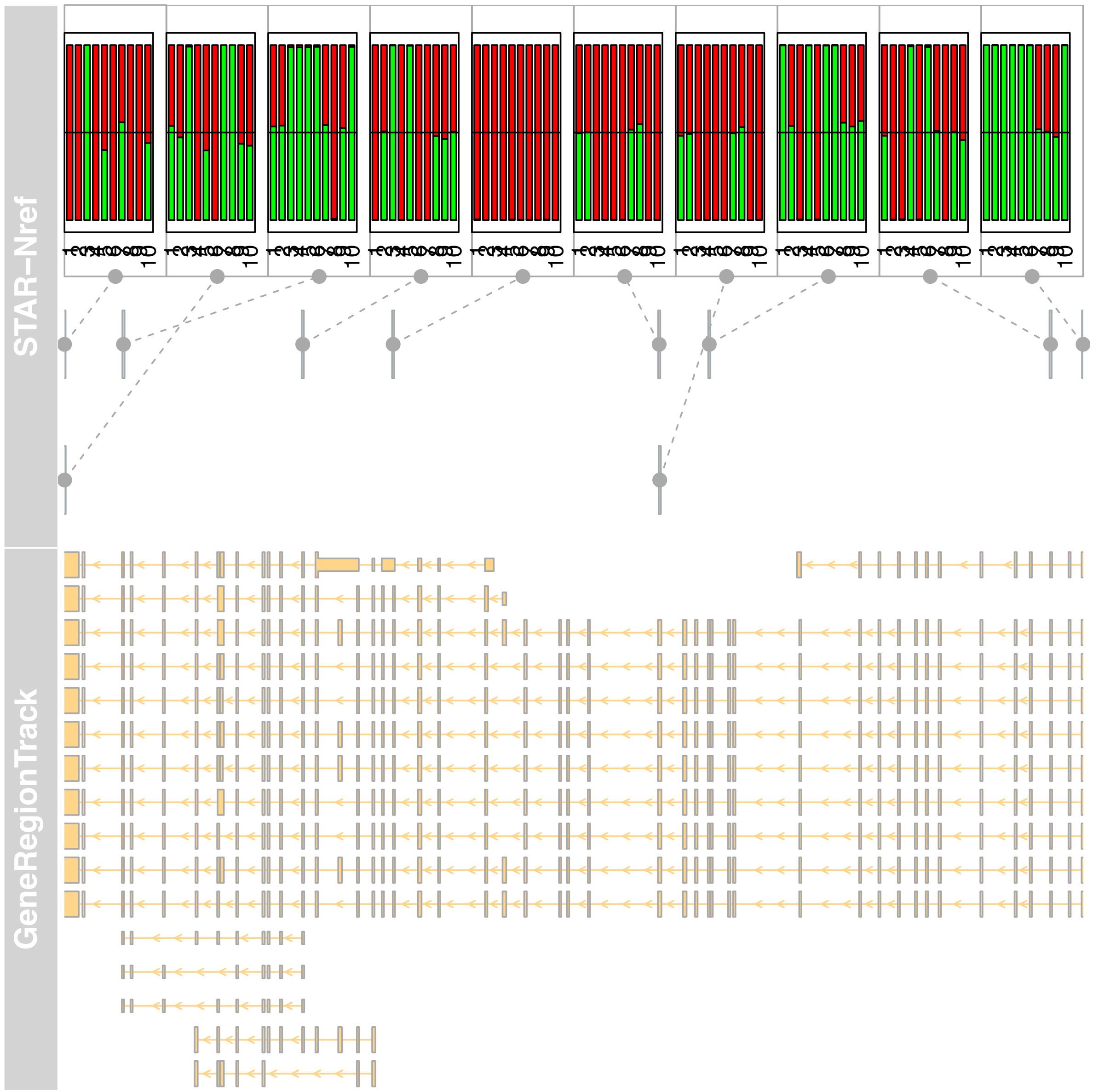

Figure A4. The *FN1* gene glocationplot with transcript information. The allele fractions are from reads mapped with the STAR aligner and dbSNP-masked reference genome.
